# Supplementary material for: High-Pressure Processing for Cold Brew Coffee: Safety and Quality Assessment under Refrigerated and Ambient Storage
Source: Foods. 2023 Nov 23;12(23):4231. doi: 10.3390/foods12234231 (PMC10705998; doi:10.3390/foods12234231)
Supplement: Supplementary file 1 [file foods-12-04231-s001.zip › foods-2663435-supplementary.pdf]

Supplementary Data

Table S1. Commercial HPP cold-brew coffee brands around the world.

Country	Company	Coffee Drinks	Website
USA	Back Bay Roasters	Black, latte	https://www.backbayroasters.com/
	Happy Tree	Blend with maple water	https://drinkhappytree.com/
	Kokomio	Blend with coconut water	https://kokomio.com/flavors/cold-brew-coffee/
	Ludwig Coffee	Black	https://ludwigcoffee.com/
	MALK Organics	Blend with pecan-based dairy alternative	https://malkorganics.com/
	Rebbl	Black, moca, blends with coconut	https://rebbl.co/
	Secret Squirrel	Black, concentrate, dark chocolate mocha, latte, Vietnamese	https://www.squirrelbrew.com/
	Tulua	Blend with date seed extract	https://www.drinktulua.com/
Indonesia	Rejuve	Latte, mocha, almond latte, almond mocha	https://www.rejuve.co.id/

Table S2. Bacterial counts expressed as Log UFC/ml of unprocessed cold brew coffee stored at 4 ºC or room temperature, and processed samples (600 MPa, 3 min) stored at 4 ºC or room temperature during 90 days of storage.

Parameter	Day of storage	Unprocessed		HPP	
		**4 ºC**	**23 °C**	**4 ºC**	**23 °C**
Total mesophilic bacteria	0	1.3 ± 0.1	1.3 ± 0.0	1.0 ± 0.1	1.0 ± 0.2
	7	1.1 ± 0.2	0.9 ± 0.1	1.0 ± 0.2	0.8 ± 0.2
	14	0.8 ± 0.1	0.9 ± 0.2	1.0 ± 0.2	0.6 ± 0.3
	28	1.3 ± 0.4	0.9 ± 0.8	0.8 ± 0.1	1.3 ± 0.5
	60	0.8 ± 0.1	0.2 ± 0.3	0.4 ± 0.4	ND
	90	1.1 ± 0.1	1.0 ± 0.6	0.7 ± 0.3	0.5 ± 0.2
Yeast and molds	0	<1	<1	1.1 ± 0.2	<1
	7	<1	<1	<1	1.1 ± 0.2
	14	<1	<1	<1	<1
	28	<1	1.1 ± 0.2	<1	<1
	60	<1	<1	<1	<1
	90	<1	<1	<1	<1
Enterobacteriaceae	0	ND	ND	ND	ND
	7	ND	ND	ND	ND
	14	ND	ND	ND	ND
	28	ND	ND	ND	ND
	60	ND	ND	ND	ND
	90	ND	ND	ND	ND

ND: not detected
